# Three Steps to Protecting Pediatric Research Participants from Excessive Risks

**DOI:** 10.1371/journal.pctr.0010025

**Published:** 2006-09-29

**Authors:** David Wendler

There is growing recognition that pediatric research is needed to improve pediatric medicine [1,2]. Research guidelines try to accommodate this need by allowing children to be enrolled in research when it offers an appropriate risk–benefit profile. These guidelines allow children to undergo research interventions that offer a compensating potential for clinical benefit. Most guidelines also allow children to undergo research interventions that do not offer a compensating potential for clinical benefit, provided the risks are acceptably low.

To implement this threshold on acceptable risks, review committees, known variously as ethics review committees, institutional review boards, or research ethics committees (RECs), must make three related assessments. They must identify the research interventions included in the study under review, determine which, if any, of the research interventions fail to offer participants a compensating potential for clinical benefit, and ensure that these interventions do not pose excessive risks.

These steps, while vital to protecting pediatric participants from excessive risks, have not been systematically described. This essay attempts to address this gap by describing the assessment appropriate for each of these three steps.

## When Is It Acceptable to Expose Children to Research Risks?

Pediatric research raises special ethical concern because children cannot provide their own informed consent. Because of children's increased vulnerability, pediatric research typically requires the permission of a parent or legal guardian. In addition, most guidelines place strict limits on the research risks to which children may be exposed.

Of greatest concern are research interventions that pose risks but do not offer participants a compensating potential for clinical benefit. These interventions pose “net” risks in the sense that the risks to participating children exceed the potential that the intervention will provide them with clinical benefit. One might argue that children should not be allowed to undergo interventions that pose net risks on the grounds that it is inappropriate to expose children to risks for the benefit of others. This approach appears to have been adopted by perhaps the first systematic research guidelines, the German guidelines of 1931, which stipulate that pediatric research shall be prohibited if it “in any way endangers the child” [3].

Prohibiting all pediatric research that poses net risks could cripple society's ability to ensure medications are safe and effective for children.

Prohibiting all pediatric research that poses net risks could cripple society's ability to ensure medications are safe and effective for children [4,5]. For example, the process of developing new medicines often requires initial pharmacokinetic studies. These studies, necessary for determining what dosage levels to use in future efficacy studies, typically do not offer a potential for clinical benefit that compensates for the risks of participating in the research. More recent guidelines aim to protect children without precluding socially valuable research by allowing children to undergo research interventions when the net risks are acceptably low. How should RECs implement this requirement?

## When Are Net Risks “Acceptably” Low?

### 

####  Leave to the judgment of the REC.

Some guidelines leave the determination of when net risks are acceptably low to the judgment of the reviewing REC (see Table 1). The Kenyan [6] and Indian Council of Medical Research [7] guidelines simply direct RECs to ensure that the risks presented by interventions that will not benefit participating children are low. The Council of Europe and the United Kingdom's Medical Research Council provide somewhat more guidance, stating that research qualifies as “minimal” risk if “it is to be expected that it will result, at the most, in a very slight and temporary negative impact on the health of the person concerned” [8,9].

**Table 1:**
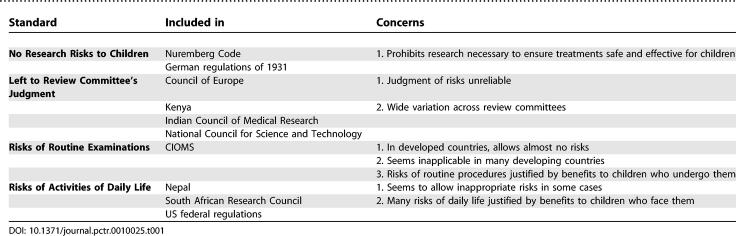
Standards for Defining Acceptably Low Risks in Pediatric Research

Mandating that risks must be low without providing a standard to make this assessment may not provide sufficient guidance. The Council of Europe's guidelines, for example, leave it to the judgment of RECs to determine when a potential harm qualifies as “slight.” Unfortunately, psychological research demonstrates that individuals make systematic errors when they rely on their own perceptions to assess risks [10–12], suggesting that RECs need more guidance.

#### Net risks no greater than risks of routine examinations.

A second approach defines acceptably low risks based on the risks of routine examinations and tests. The Council for International Organizations of Medical Sciences (CIOMS) takes this approach, deeming research interventions acceptable when the risks “do not exceed those associated with routine medical and psychological examination of such persons” [13]. Similarly, the Canadian Tri-Council report states that risks qualify as minimal when potential participants can reasonably be expected to regard them as “no greater than those encountered by the subject in those aspects of his or her everyday life that relate to the research” [14].

Although the Canadian definition is not as clear as one might hope, both standards appear to allow patients who undergo risky examinations as part of their medical care to be exposed to greater research risks than healthy children. To avoid taking advantage of sick children in this way, several commentators define minimal risks based on the risks posed by routine examinations for *healthy* children [15,16].

The Bright Futures guidelines, endorsed by the American Academy of Pediatrics (AAP), recommend that healthy children be assessed for height, weight, head circumference, vision, and hearing [17]. The only invasive examination recommended for healthy children is a single heel stick at birth to screen for metabolic disorders. In practice, then, the healthy-child version of the routine examinations standard avoids the potential to exploit sick children by essentially precluding research that poses any net risks. Furthermore, children in some countries do not undergo routine medical examinations, leaving it unclear how this standard would be applied in those countries.

#### Net risks no greater than risks of daily life.

A third approach defines acceptably low risks based on the risks children face in daily life. Australia's guidelines categorize research interventions as posing minimal risk when “the probability and magnitude of harm or discomfort anticipated in the research are not greater in and of themselves than those ordinarily encountered in daily life” [18]. Guidelines from Nepal [19] and the United States [20,21] combine this definition with the routine examinations standard, defining minimal risks as “not greater in and of themselves than those ordinarily encountered in daily life or during the performance of routine physical or psychological examinations or tests.”

The “risks of daily life standard” would seem to allow children in war-torn areas to be exposed to enormous risks for the benefit of others. Recognizing this concern, the South African Medical Research Council limits the “risks of daily life” standard to the risks “normally encountered in the daily lives of people in a stable society” [22].

The risks of daily life standard provides an objective comparator for evaluating the risks of pediatric research, thus avoiding the pitfalls of relying exclusively on the individual judgment of REC members. To implement this standard effectively, RECs must distinguish the called-for comparison from the comparisons appropriate to the other two steps in the risk assessment process.

## Clarifying the Comparisons Involved in the Risk Assessment Process

### 

####  Step 1: Identifying the research interventions.

**Figure 1:**
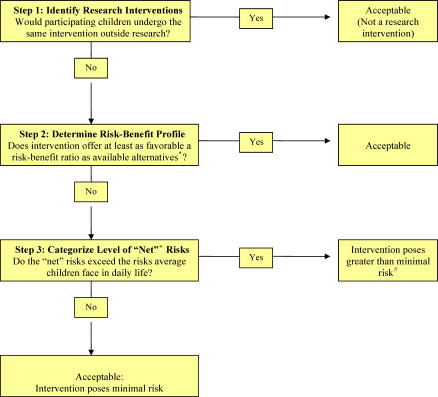
Step Assessment of the Risks of Pediatric Research Interventions *, including not undergoing the intervention at all; +, “net” risks are determined by the extent to which the risks exceed the potential for clinical benefit; #, some regulations allow RECs to approve greater than minimal risk interventions when the risk is no more than a minor increase over minimal and the study meets several other conditions.

Clinical studies often involve both research and standard-of-care interventions. To minimize the number of interventions they need to review, RECs can first determine which interventions qualify as research interventions (see Figure 1). The US federal regulations, for example, direct RECs to evaluate the risks of only the research interventions, as opposed to any “therapies subjects would receive even if not participating in the research.”

This approach is based on the assumption that any interventions participating children otherwise would undergo as part of their routine clinical care have been determined to be appropriate for them. Thus, RECs need assess only those interventions that are not part of the participating children's routine clinical care.

Tragically, children in developing countries often do not receive any routine medical care, including proven effective treatments. As a result, an intervention may qualify as a research intervention when used in a developing country, but not when it is part of a study in a country where children would receive the intervention even if they did not participate in research. While initially puzzling, this difference makes sense. Standard treatments not routinely provided in a particular setting are likely not to have been assessed for efficacy and toxicity in that setting. Hence, it makes sense for the reviewing REC to assess these interventions carefully, even though they represent routine medical care in other countries, and may not need to undergo REC review and approval.

#### Step 2: Determining the risk–benefit profile of the research interventions.

The risks and benefits posed by research interventions can vary depending on the children who undergo them. The risks of an MRI scan are very different for children who suffer from panic disorders compared with healthy children. Since RECs must determine the risk–benefit profile of pediatric research procedures prospectively, they cannot make this determination for the specific children who will be enrolled. Instead, the REC should assess the risk–benefit profile of each research intervention for the children eligible for the study.

If the risk–benefit profile of some interventions varies significantly by different groups of children who are eligible, the REC should determine the risk–benefit profile for each of the distinct groups. If the REC finds that one or more groups faces significantly higher risks compared with other eligible children, it should consider excluding those who face greater risks. Exclusion of children who face significantly higher risks makes sense especially when their participation is not vital for scientific reasons, and the study does not offer them an important potential for clinical benefit that is unavailable outside the research context.

The fact that a research intervention offers participating children a potential for clinical benefit that compensates for its risks does not imply that the intervention is necessarily acceptable. In some cases, the treatments participants receive in research replace treatments they otherwise would receive in the clinical setting. When this happens, RECs should assess whether the research treatment offers a risk–benefit profile that is at least as favorable as the available alternatives, including not undergoing the intervention at all.

To assess an intervention's risk–benefit profile, RECs should compare it with the risk–benefit profile of the available alternatives for the participating children. It follows that a given research treatment may have a favorable risk–benefit ratio when administered in a developing country to children who have no available alternatives, but an unfavorable risk–benefit ratio in a developed country that provides children clinically indicated treatment.

A number of authors have pointed out that allowing some interventions to be tested in developing countries but not in developed countries raises the potential for exploitation [23–25]. This potential for exploitation could be addressed by insisting that the risk–benefit profile of research interventions be compared with the risk–benefit profile of the best interventions available anywhere in the world. This solution implies that an experimental treatment studied in a developing country that offers a compensating potential for clinical benefit that is better than any alternatives available there nonetheless poses an unacceptable risk–benefit ratio if the treatment is not as good as the treatments used in the developing world.

This approach has the potential to deny deprived children effective treatments in the name of protecting them. A better approach would be to recognize that the concern posed by such trials is not the risk–benefit profile of the research interventions, but the potential to exploit individuals' deprived circumstances. While beyond the scope of the present paper, safeguards have been proposed to address this type of exploitation without altering the process of risk assessment [26].

When the risk–benefit profile of a research intervention is at least as favorable as the available alternatives, the intervention poses no net risks—hence, it offers an ethically acceptable risk–benefit profile. If the risk–benefit profile of the intervention is less favorable than one or more of the available alternatives, it poses “net” risks. The magnitude of an intervention's net risks is determined by the extent to which it presents increased risks, or a decreased potential for clinical benefit compared with the available alternatives, including not undergoing the intervention at all.

#### Step 3: Assessing the “net” risks.

The “risks of daily life” standard directs RECs to assess the net risks of research interventions by comparing them with the level of risk ordinarily encountered in daily life by average healthy children. At this step in the risk assessment process, the appropriate comparison is between the risks the specific participants face from the research interventions and the risks average children face in daily life (or during the performance of routine examinations).

Importantly, to assess net risks, RECs should not compare the interventions included in the research with the interventions the same children undergo as part of their routine medical care. For example, the fact that some children routinely undergo a particular type of biopsy as part of their clinical care does not imply that the risks of that type of biopsy are necessarily minimal for them. In contrast, to determine which interventions qualify as research interventions in the first place, RECs do compare the interventions included in the study under review with the interventions *the same children* would receive even if they did not participate in research.

#### Example: Randomized trial of new malaria treatment.

To illustrate the three-step risk assessment method, consider how it would apply to a study of a new treatment for childhood malaria. Imagine that the study provides all participants with standard-of-care treatment, and then randomizes half of the participants to also receive an experimental, add-on treatment. All participants undergo a series of blood draws, some for clinical care and a few for research purposes. In addition, the researchers propose to take a minimal amount of extra blood for research purposes during several of the clinically indicated blood draws.

##### Step 1:

To assess the risks of this study, the REC first should determine which of the interventions qualify as research interventions, by comparing the study interventions with the clinically indicated interventions the same children would receive even if they did not participate in research. Since clinically indicated treatment includes the standard medications and the clinically indicated blood draws, these interventions do not qualify as research interventions. Because participants would not receive the experimental, add-on treatment, or undergo the research blood draws in the clinical setting, these interventions qualify as research interventions. For the same reason, the collection of extra blood during the clinically indicated blood draws qualifies as a research intervention.

###### Step 2:

In the second step, the REC should assess the risk–benefit profile of each of the research interventions for the groups of children who are eligible for the study. Whether the add-on treatment can be categorized as offering a favorable risk–benefit ratio will depend on the nature and extent of previous experience with the drug. At this step, the REC should consider all of the previous experience with the drug, including whether it has been used to treat other conditions, whether it has been used in adults, and whether it has ever been used in children with malaria. In each case, the REC should assess to what extent the previous experience is relevant to the current study, given the children who will be enrolled, the doses to be used, and the other medications that will accompany the new treatment.

The add-on treatment may have a synergistic effect, providing greater benefit in combination with the other treatments. Alternatively, combining it with other medications may create a greater potential for toxicity compared with the use of the new treatment as a single agent. Taking these possibilities into account, the REC should determine whether the experimental treatment offers a favorable risk–benefit ratio to participating children and, if so, whether its risk–benefit profile is at least as favorable as the available alternatives. If it is, the treatment can be approved as offering a compensating potential for medical benefit. If the treatment offers a risk–benefit profile less favorable than the available alternatives, including not undergoing the intervention at all, it poses net risks.

Presumably, the research blood draws and the extra blood drawn during the clinically indicated blood draws do not offer the potential for clinical benefit. Thus, the net risks of these interventions are represented by all the risks they pose to the participating children, taking into account their overall clinical condition, as well as how much total blood will be drawn during the study period.

####### Step 3:

To assess the acceptability of an intervention's net risks under the risks of daily life standard, the REC should compare the net risks that the intervention poses to the children in the study with the level of risks average children face in daily life (or during routine examinations). Notice that comparing the interventions the children receive in the study with the interventions these same children receive routinely as part of their clinical care raises the potential to exploit those children who face greater risks in their daily lives. For example, the REC should not categorize the extra blood draws as minimal risk simply on the grounds that the participating children happen to receive many blood draws as part of their standard medical care. Indeed, the fact that some children receive many blood draws as part of their standard medical care may well increase the risks of the research blood draws, for example, by increasing the chances that the research blood draws will induce anemia in these children.

## Conclusions

Evaluation of the risks of pediatric research involves several steps, each of which requires a slightly different comparison. Clarifying the comparison appropriate to each step should help RECs to protect children and ensure that advances in pediatric medicine are not gained at the cost of exploiting some children.

Because many, perhaps most interventions in the research setting pose risks yet offer some potential for clinical benefit, the concept of net risks is central to the risk assessment process. This concept is familiar to clinicians who often must assess whether the potential benefits of a given treatment justify its risks. However, in the clinical setting, interventions are considered inappropriate if they pose any net risks at all. In the research setting, net risks may be acceptable if they are sufficiently low. As a result, RECs must make the more complicated determination of estimating the magnitude of the net risks of a given intervention. Future research should consider different approaches that RECs might use to estimate the magnitude of net risks posed by research interventions.

## Abbreviations

REC: research ethics committee
